# A “Refuse” Case

**Published:** 1894-03

**Authors:** J. B. Campbell

**Affiliations:** Brooklyn, N. Y.


### A "REFUSE" CASE.

J. B. CAMPBELL, M. D., BROOKLYN, N. Y.

Homœopathists are constantly meeting with physical "derelicts" who have been abandoned to their fate by the scientific (?) medical fraternity. It was to one of these that I was called in June, 1892, Harrison K., aged four years, suffering from marasmus, who had been given up to die by two "regulars." The child, a miserable, scrawny specimen, lay on a pillow moaning with pain and scarcely able to move, so great was the prostration—mental irritability very marked; emaciation extreme, the skin on inside of thighs hanging in flabby folds; complete anorexia; considerable meteorism, the abdomen being hard, distended, and tympanitic; sore spot in left hypochondrium; sensitive to pressure; perspiration general, but more marked about head and neck; stools scanty, light-colored, and offensive, containing undigested food, mucus, and blood.

A single dose of Calc-carb.$^{45m}$ produced a change for the better at once, slowly at the start, but more pronounced after the first week; Sac-lac. for one month, after which the case seemed to come to a standstill as concerned further improvement.

Another dose of Calc.$^{cm}$, followed by S. L. for three weeks, completed the cure, after which time the child was playing about as usual, to the amazement of relatives, neighbors, and even the "regular" M. D.'s; but any Hahnemannian homœpath would have wondered, not at this particular cure, which is only one instance of many, but at the marvelous and unerring precision with which God's eternal law manifests His watchful presence, a benefit to the suffering, and a reward to those who seek and abide by the truth.

